# Network Pharmacology and Molecular Docking to Explore the Mechanism of Kangxian Decoction for Epilepsy

**DOI:** 10.1155/2022/3333878

**Published:** 2022-09-23

**Authors:** Weitao Wang, Yongquan Zhang, Yibing Yang, Lian Gu

**Affiliations:** ^1^Guangxi University of Chinese Medicine, Nanning 530200, China; ^2^Ruikang Hospital Affiliated to Guangxi University of Chinese Medicine, Nanning 530011, China; ^3^The First Affiliated Hospital of Guangxi University of Chinese Medicine, Nanning 530023, China

## Abstract

**Purpose:**

Kangxian decoction (KXD) has been used in clinical practice to treat epilepsy. The purpose of this study was to explore the active components of KXD and clarify its antiepileptic mechanism through network pharmacology and molecular docking.

**Methods:**

The components of KXD were collected from the Encyclopedia of Traditional Chinese Medicine (ETCM) database and the literature was searched. Then, active ingredients were screened by SwissADME and potential targets were predicted by the SwissTargetPrediction database. Epilepsy-related differentially expressed genes were downloaded from the Gene Expression Omnibus database. A component-target-pathway network was constructed with Cytoscape. Kyoto Encyclopedia of Genes and Genomes (KEGG) enrichment analysis and protein‒protein interaction network analysis revealed the potential mechanism and critical targets. Receiver operating characteristic (ROC) curves and box plots in microarray data validated the good diagnostic value and significant differential expression of these critical genes. Molecular docking verified the association between active ingredients and essential target proteins.

**Results:**

In our study, we screened the important compounds of KXD for epilepsy, including quercetin, baicalin, kaempferol, yohimbine, geissoschizine methyl ether, baicalein, etc. KXD may exert its therapeutic effect on epilepsy through the following targets: PTGS2, MMP9, CXCL8, ERBB2, and ARG1, acting on the following pathways: neuroactive ligand-receptor interactions, arachidonic acid metabolism, IL-17, TNF, NF-kappa B, and MAPK signaling pathways. The molecular docking results showed that the active ingredients in KXD exhibited good binding ability to the key targets.

**Conclusion:**

In this study, we explored the possibility that KXD for epilepsy may act on multiple targets through multiple active ingredients, involving neurotransmitters and neuroinflammatory pathways, providing a theoretical basis for subsequent clinical and experimental studies that will help develop effective new drugs to treat epilepsy.

## 1. Introduction

Epilepsy is considered one of the most common serious brain disorders and affects more than 70 million people worldwide. It is not a disorder with a single presentation or cause, but a combination of symptoms with multiple risk factors and a strong genetic predisposition. Epilepsy has a bimodal distribution of prevalence, with infants and older populations at the highest risk [1]. Anxiety, depression, peptic ulcers, dementia, heart disease, and migraines are eight times more common in people with epilepsy than in the general population, leading to a high burden of comorbidities in people with epilepsy [2]. Approximately 80% of people with epilepsy live in low- and middle-income countries. The burden on people with epilepsy can be reduced through effective treatments [3]. However, although more than 20 antiepileptic drugs can be used for symptomatic treatment of seizures, approximately 1/3 of epileptic patients have seizures that are ineffective to drug therapy [4].

Traditional Chinese medicine (TCM) has been used for thousands of years as a unique treatment modality with the advantages of clinical efficacy, few side effects, and low drug resistance. In recent years, TCM research as a new treatment strategy has gained increased attention. Chinese medicine has a long history of treating epilepsy. Different herbs can exert antiepileptic effects through various mechanisms such as enhancing GABAergic effects, modulating sodium and NMDA channels, and exerting antioxidant, anti-inflammatory, and neuroprotective properties [5].

Kangxian decoction (KXD) is composed of six traditional Chinese medicines, including *Acorus tatarinowii* (Chinese name: Shichangpu), *Pinellia ternata* (Chinese name: Banxia), *Panax notoginseng* (Chinese name: Sanqi), *Gastrodia elata* (Chinese name: Tianma), *Uncaria* (Chinese name: Gouteng) and *Pheretima* (Chinese name: Dilong), which have the effect of eliminating phlegm and promoting blood circulation. The names of the plants involved are consistent with “The Plant List” (https://www.theplantlist.org/). Previous clinical and experimental studies have shown that KXD significantly prolongs the seizure incubation period, reduces seizure level, duration, and number of seizures in epileptogenic rats, and achieves antiapoptotic effects by reducing hippocampal Glu content, increasing GABA content, and promoting Bcl-2 protein expression [6–9]. In addition, traditional Chinese medicine relies on multicomponent, multitarget, and multipathway methods to treat complex diseases that are too complicated to analyze by conventional experimental methods. Therefore, we need innovative methods to study the pharmacological mechanism of KXD. As a new method, network pharmacology, combined with system network analysis and pharmacology, can clarify the synergistic effect and potential mechanism of the compound-target-disease network at the molecular level [10, 11]. This is consistent with the multicomponent, multitarget, and multipathway mechanism of action of TCM compounds and is in line with the principles of the holistic view and dialectical treatment in TCM. In particular, the application of network pharmacology in TCM provides researchers with a novel opportunity to understand TCM systematically, which may create new directions for studying potential pharmacological mechanisms and safety evaluation of TCM [12]. Molecular docking refers to the spatial docking of a small molecule into a macromolecule and obtaining complementary values for the binding site, which is used for structure-based drug design [13]. At present, although some herbal medicines are used to treat epilepsy, there is a lack of systematic and comprehensive observation of the pathways of drug intervention in the disease network. Therefore, this study investigated the molecular mechanism of action of KXD in the treatment of epilepsy using network pharmacology and molecular docking, which will provide a reference for future pharmacological and clinical studies and help develop new effective drugs for the treatment of epilepsy.

## 2. Materials and Methods

### 2.1. Screening of Active Ingredients and Potential Targets in KXD

The components in the KXD were collected from the Encyclopedia of Traditional Chinese Medicine (ETCM) database (https://www.tcmip.cn/ETCM/) [14]. To ensure the comprehensiveness and accuracy of the data, we also identified reported active ingredients from the PubMed, Web of Science, and China National Knowledge Infrastructure (CNKI) databases. The collected chemical components were individually entered into the PubChem database (https://pubchem.ncbi.nlm.nih.gov/) [15] to obtain the CAS number and canonical SMILES. Canonical SMILESs of the acquired compounds were put into the SwissADME platform (https://www.swissadme.ch/) [16]. The screening of the active ingredients was based on the following characteristics: (i) gastrointestinal absorption (GI absorption) was “High,” indicating good oral bioavailability and absorption of the ingredient; (ii) at least two of the five categories (Lipinski, Ghose, Veber, Egan, Muegge) were set to “Yes,” indicating that the compound has good drug-like properties [17]. Although some of the ingredients did not satisfy the above criteria, they were still included if their good pharmacological properties were confirmed through a literature review. Finally, the canonical SMILES of the collected active ingredients were entered into the SwissTargetPrediction database (https://www.swisstargetprediction.ch/) [18] to obtain the corresponding target genes. The criterion for target screening was a probability greater than 0.1.

### 2.2. Identification of Differentially Expressed Genes

The expression profile data of GSE143272 and the microarray platform GPL10558 were downloaded from the GEO database (https://www.ncbi.nlm.nih.gov/geo/). The expression profile data contained 34 untreated epilepsy patient samples (blood) and 51 normal samples (blood). Differentially expressed genes (DEGs) from the epilepsy samples and normal samples were screened using the limma package in R 4.0.2 software based on |logFC|>0.3, adj. *P* value < 0.05. Visualization of DEGs was performed using the ggplot2 package as a volcano plot. From intersecting drug target genes with DEGs using the VennDiagram package, we eventually obtained KXD and epilepsy common associated genes.

### 2.3. Constructing Protein‒Protein Interaction (PPI) Networks and Screening Key Genes

KXD and epilepsy common-related genes were used to construct the PPI network using the STRING database (https://string-db.org/cgi/input.pl) [19]. The PPI network was obtained with default settings. The PPI network from the STRING database was then imported into Cytoscape 3.7.2 to research the critical genes. To ensure the accuracy of the results, we used two different plugins of Cytoscape to obtain two key subnetworks separately. First, using the MCODE plug-in in Cytoscape, the PPI network was further analyzed to obtain the potential module. Another way to retrieve the key subnetwork was to use the cytoHubba plug-in and apply the MCC algorithm to analyze the top 5 genes in the PPI network to obtain the second key subnetwork. Finally, we screened critical genes from the two subnetworks.

### 2.4. Receiver Operating Characteristic (ROC) Curves and Box Plots for Hub Genes

To assess the diagnostic ability of each hub gene for epilepsy, ROC curves were plotted, and the area under the curve (AUC) values were calculated using the pROC package in R software. The differential expression of hub genes between normal and epilepsy samples was assessed using box plots.

### 2.5. Pathway Enrichment Analysis

The cluster profiler package in R software was used to perform the Kyoto Encyclopedia of Genes and Genomes (KEGG) pathway enrichment analysis of genes commonly associated with KXD and epilepsy. *P* < 0.05 was considered significant.

### 2.6. Constructing the Component-Target-Pathway Network

Cytoscape was used to build a network of relationships between active ingredients, common targets for KXD and epilepsy, and pathways. In the component-target-pathway network, “nodes” represent components, targets, and pathways, and “edges” represent the relationships between them. The “degree” parameter represents the number of connections between nodes in the network and is used to assess the important components and targets.

### 2.7. Molecular Docking

**Figure 1:**
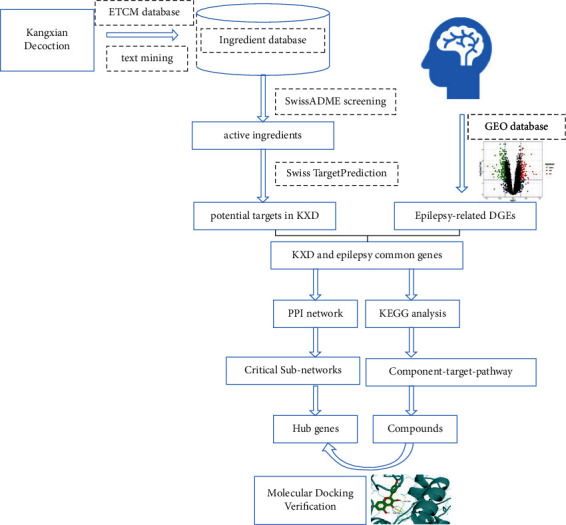
Workflow of the entire network pharmacology and molecular docking.

To validate the component-target association, the key target genes were selected for subsequent molecular docking analysis. The three-dimensional (3D) structure of the target protein was downloaded from the RCSB PDB database (https://www.rcsb.org/), and the two-dimensional structure of the molecular ligand was downloaded from the PubChem database (https://pubchem.ncbi.nlm.nih.gov/). The proteins and molecules were preprocessed with PyMOL 2.4.0 software and saved in pdb format. The AutoDock Tools 1.5.6 hydrogenated and calculated the charges, selected the proteins as receptors and the molecular compounds as ligands, and then set up the docking parameters. The Grid Box was used to generate the docking area, which was defined as a 40 × 40 × 40 three-dimensional grid centered on the ligand binding site, with a grid space of 0.375 Å. Finally, the docking of the receptor protein and the molecular ligand was completed using AutoDock Vina 1.1.2, and the binding energy was obtained. The optimal conformation was selected and visualized using PyMOL. The entire network pharmacology and molecular docking process are illustrated in Figure 1.

## 3. Results

### 3.1. Screening of Active Ingredients and Potential Targets in KXD

After SwissADME screening and removing some compounds without targets, 49 active ingredients from six herbs were identified in the KXD formulation. Including 6 from *Acorus tatarinowii* (Chinese name: Shichangpu), 11 from *Pinellia ternata* (Chinese name: Banxia), 10 from *Panax notoginseng* (Chinese name: Sanqi), 8 from *Gastrodia elata* (Chinese name: Tianma), 18 from *Uncaria* (Chinese name: Gouteng), and 6 from *Pheretima* (Chinese name: Dilong). Notably, some of these ingredients were present in at least two herbs. Details of the active ingredients are shown in Table 1.

According to the target screening, there were 149 targets in Shichangpu, 212 targets in Banxia, 292 targets in Sanqi, 159 targets in Tianma, and 517 targets in Gouteng in KXD. After merging and removing duplicates, 705 target genes corresponding to the 49 active ingredients in KXD were finally identified.

### 3.2. Identification of Epilepsy-Related DEGs

**Figure 2:**
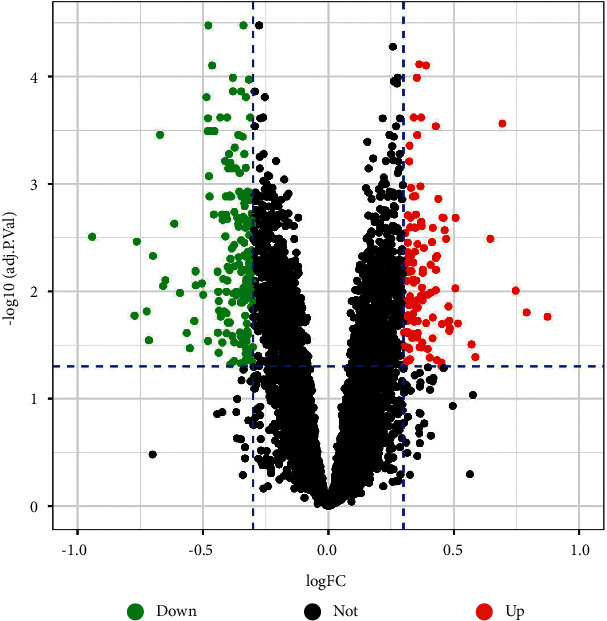
Volcano plot of differentially expressed genes, with red dots representing significantly upregulated genes, green dots representing significantly downregulated genes, and black dots representing not significant.

**Figure 3:**
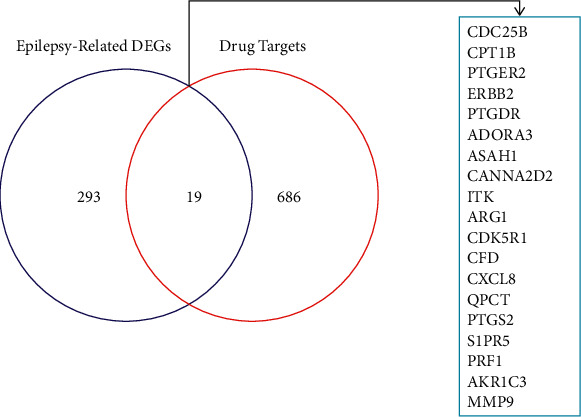
Venn diagram showing commonly related genes in KXD and epilepsy.

We defined the filtering criteria as |logFC|>0.3, adj. *P* value < 0.05. Finally, 312 differentially expressed genes associated with epilepsy were obtained, including 189 upregulated and 123 downregulated genes, and the results are presented in a volcano plot (Figure 2). For intersecting drug target genes with DEGs, we eventually obtained 19 genes commonly associated with KXD and epilepsy. The results are shown in a Venn diagram (Figure 3).

### 3.3. Construction of a Network of Component-Target-Pathway

**Figure 4:**
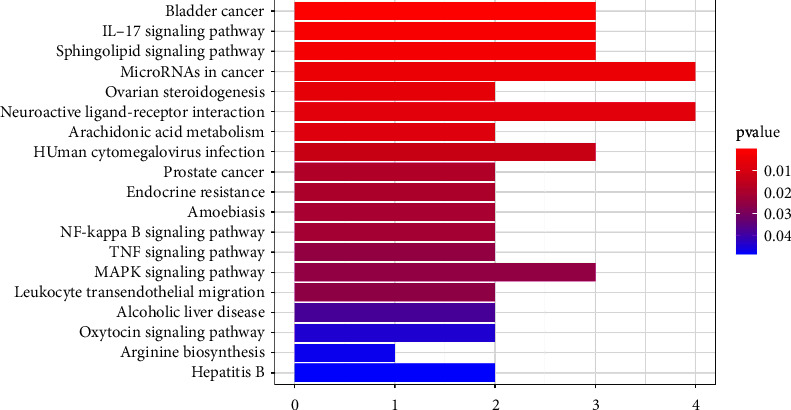
KEGG enrichment analysis of common target genes.

**Figure 5:**
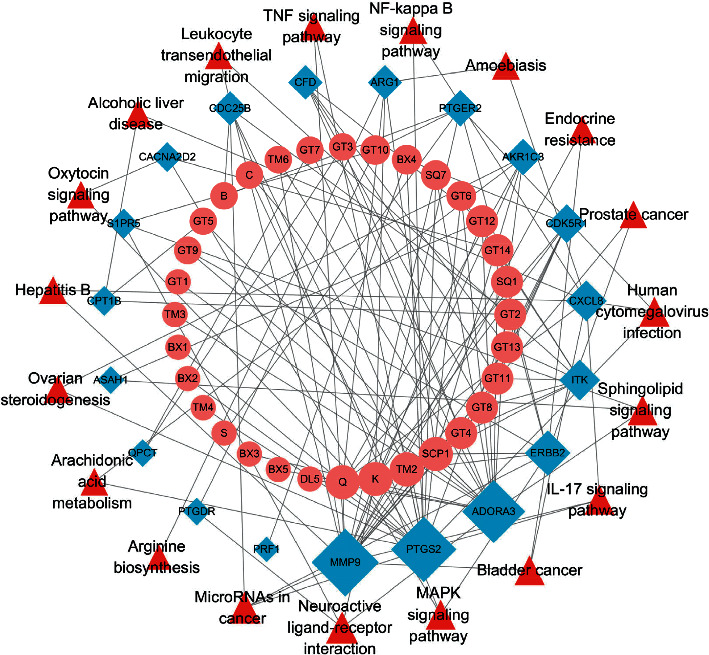
Network of component-target-pathway. The circles represent the ingredients, the rhombuses represent target genes, and the triangles represent pathways.

To further understand the crossed genes, we performed a KEGG enrichment analysis. A total of 19 KEGG pathways were screened based on *p* values < 0.05 (Figure 4). Details of the KEGG enrichment analysis can be found in Table 2. Major pathways included the sphingolipid signaling pathway, arachidonic acid metabolism, neuroactive ligand-receptor interaction, IL-17, TNF, NF-kappa B, and MAPK signaling pathways. We also identified 32 active ingredients targeting 19 intersecting targets from all active ingredients of KXD. To investigate the association between components, targets, and pathways, we constructed a component-target-pathway network (Figure 5). In general, a single ingredient can target multiple target genes, and a single target can be affected by multiple ingredients. The larger the area is, the larger the degree value, meaning the more important it is likely to be in the network. The network diagram showed that the mechanism of action of KXD has multicomponent, multitarget, and multipathway characteristics.

### 3.4. Identification of Hub Genes and Important Components

**Figure 6:**
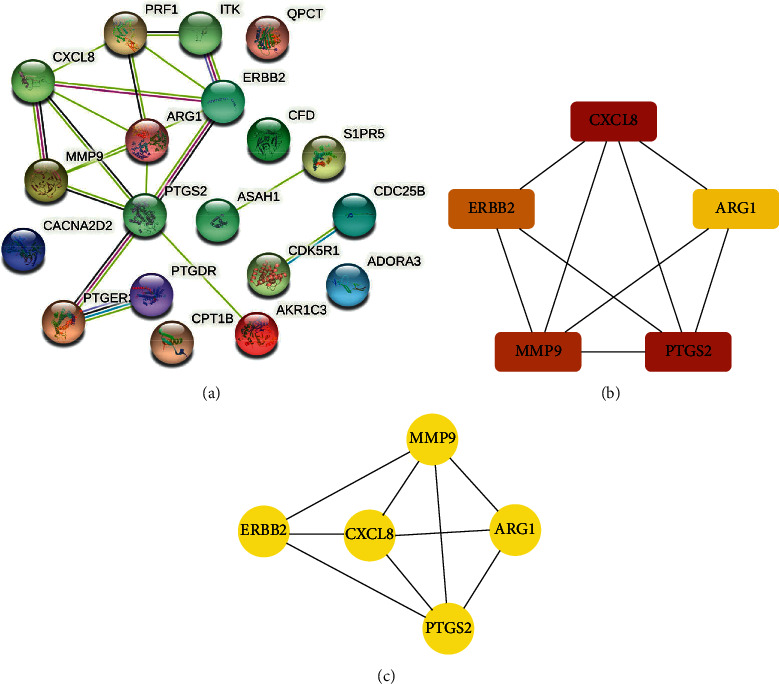
Identification of hub genes. (a) PPI protein interaction network downloaded from the STRING database. (b) Hub genes obtained using cytoHubba. (c) Hub genes obtained using MCODE.

The 19 diseases and drug common target genes were imported into the STRING database, and tsv files were downloaded and imported into Cytoscape to construct PPI networks. Using the cytoHubba and MCODE plugins, we screened two identical subnetworks containing five hub genes, including MMP9, PTGS2, CXCL8, ERBB2, and ARG1 (Figures 6(a)–6(c)). We also identified 21 active ingredients targeting these hub genes through the component-target-pathway network. Based on literature reports of proven antiepileptic pharmacological effects, we screened six important components and validated them for subsequent molecular docking. These components were kaempferol, quercetin, baicalein, baicalin, yohimbine, and geissoschizine methyl ether.

### 3.5. Verification of Hub Genes Using ROC Curves and Box Plot Analysis

**Figure 7:**
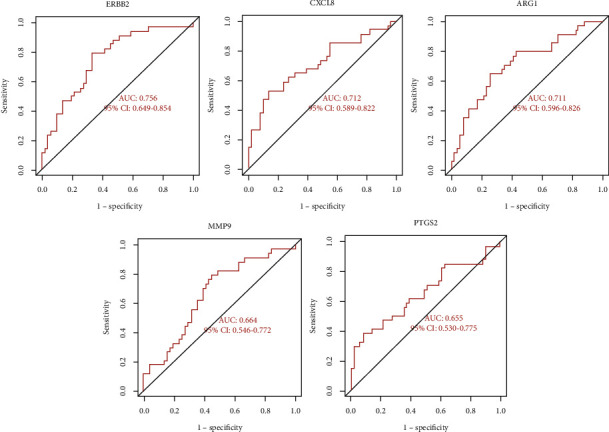
The hub genes were validated in the GSE143272 dataset using ROC curves. AUC represents the area under the curve.

**Figure 8:**
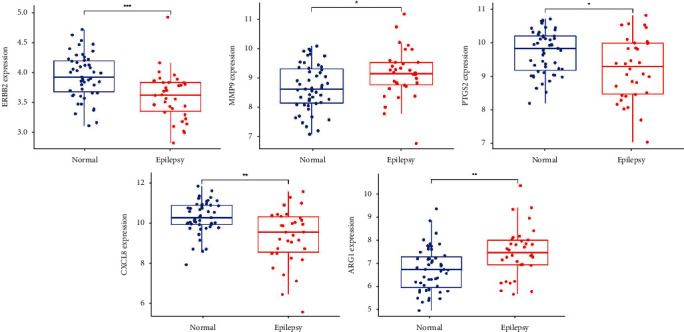
Hub genes were verified in the GSE143272 dataset using box plots. Box plots indicated that all five hub genes were differentially expressed in the microarray dataset. ^*∗*^*P* < 0.05, ^*∗∗*^*P* < 0.01, and ^*∗∗∗*^*P* < 0.001.

ROC curves were applied to evaluate the diagnostic value of the hub genes for epilepsy in the GSE143272 dataset. The AUC values of PTGS2, MMP9, CXCL8, ERBB2, and ARG1 were all greater than 0.65, indicating a good diagnostic value (Figure 7). Box plots indicated that all five hub genes were differentially expressed in the microarray dataset (Figure 8).

### 3.6. Molecular Docking Results

**Figure 9:**
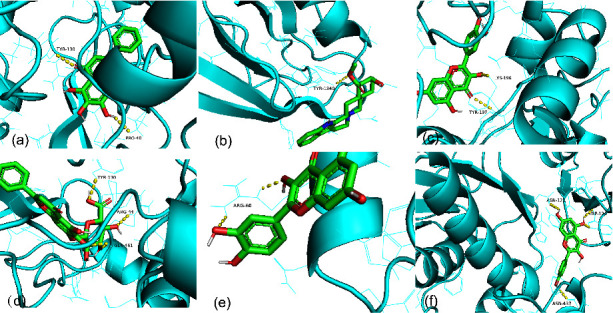
The conformations of certain important compounds and key targets. (a) Baicalein with PTGS2. (b) Yohimbine with ERBB2. (c) Quercetin with ARG1. (d) Baicalin with PTGS2. (e) Quercetin with CXCL8. (f) Kaempferol with MMP9.

The five hub genes were selected for molecular docking analysis. Molecular docking was used to verify whether certain important compounds had a significant role in the regulation of the key target proteins. These important compounds were quercetin, baicalin, kaempferol, yohimbine, geissoschizine methyl ether, and baicalein. The interactions between the optimized conformations of certain important components and the key targets are shown visually in Figure 9. The docking scores are shown in Table 3. A binding energy of less than “−5” means that the compound and the target are well bound to each other [20]. The highest docking score was obtained for PTGS2 with baicalin at -10.1 kcal·mol^−1^ and the lowest docking score was obtained for CXCL8 with quercetin at -5.3 kcal·mol^−1^. Molecular docking showed that both these active compounds and the target proteins have good binding properties.

## 4. Discussion

Pharmaco-resistance to antiepileptic drugs (AEDs) remains a primary unresolved therapeutic issue. Different antiepileptic drugs have been reported to cause serious side effects, with the development of pharmaco-resistance [21]. KXD is a Chinese herbal formula for the treatment of epilepsy, the effectiveness of which has been shown in our previous clinical research [6, 22, 23]. In our study, we screened the important compounds of KXD for epilepsy, including quercetin, baicalin, kaempferol, yohimbine, geissoschizine methyl ether, baicalein, etc.

Quercetin and kaempferol, both phytoflavonoids, are important dietary components of fruits and vegetables and have various biological effects, including antioxidant, anti-inflammatory, and neuroprotective activities. In vivo studies have shown that these phytoflavonoids can modulate the immune response, becoming an important treatment alternative for epilepsy [24]. GABA exists widely in the mammalian brain and is the main inhibitory neurotransmitter. Quercetin pretreatment exerted an inhibitory effect on GABAA receptor gene expression in a KA epilepsy model [25]. Silva et al. summarized the therapeutic effects of kaempferol in central nervous system disorders over the past ten years and found that kaempferol has significant anti-inflammatory pharmacological effects and exerts antioxidant effects through inhibition of MMP9 metalloproteinases, exhibiting multipotential neuroprotective effects [26]. The component-target-pathway network in this study also revealed that kaempferol may exert anti-inflammatory and neuroprotective effects by targeting MMP9. Yohimbine is a potent *α*2-adrenoceptor antagonist with anticonvulsant effects at low doses [27]. Geissoschizine methyl ether is a promising antiepileptic drug obtained from indole alkaloids isolated from Uncaria that can inhibit multiple neuronal channels [28]. Baicalein, as an important flavonoid, has various pharmacological effects, including anti-apoptotic, anti-inflammatory, antioxidant, and neuroprotective activities [29, 30]. Studies have demonstrated the neuroprotective effect of baicalin on PTZ-induced seizures [31].

The PPI network identified PTGS2, MMP9, CXCL8, ERBB2, and ARG1 as key genes. Inflammatory pathways play an essential role in the pathophysiology of epilepsy [32]. PTGS2, also known as COX2, is a major proinflammatory mediator associated with the severity and recurrence of epilepsy [33]. Epileptogenesis is associated with increased matrix metalloproteinases (MMPs) [34]. Tao et al. found that serum levels of MMP9 were higher in epileptic patients than in normal individuals and may serve as a biomarker to differentiate the cause of epilepsy [35]. This is consistent with the finding in this study that MMP9 was differentially expressed in the microarray dataset. Research has demonstrated that the CXCL8 (IL-8) signaling pathway can be activated during seizures [36]. ARG1 deficiency can lead to seizures, neurodevelopmental disorders, etc. [37]. Lamie et al. found that ERBB2 inhibitors could be a potential anticonvulsant drug [38]. These 5 key genes also demonstrated good diagnostic ability in the dataset using ROC curves and box plots.

Neuroinflammation-related pathways could be biomarkers and important therapeutic targets for epilepsy [39]. Glutamate and gamma-aminobutyric acid (GABA) are the main neurotransmitters that play key roles in the pathophysiology of epilepsy [40]. Numerous studies have shown that inflammatory responses and neurotransmitters in the brain play an important pathophysiological role in epilepsy [39, 40]. KEGG enrichment analysis enriched a total of 19 pathways involving neurotransmitters, including neuroactive ligand-receptor interactions and inflammatory responses, including arachidonic acid metabolism and the IL-17, TNF, MAPK, and NF-kappaB signaling pathways. Research has shown that abnormal interactions between neuroactive ligands and receptors can increase susceptibility to seizures [41]. Epileptogenesis is a complex pathological process involving mainly inflammatory responses and neuronal death [42]. The NF-*κ*B pathway is associated with inflammation and oxidative stress, plays a key role in epileptogenesis, and can regulate neuronal survival and apoptosis [43]. The IL-17 signaling pathway and TNF signaling pathway play a critical role in inducing blood-brain barrier disruption and CNS autoinflammation, which are closely associated with seizures [44]. Cyclooxygenase (COX) converts arachidonic acid into prostaglandins, and activation of COX-2 is thought to be a trigger for neuroinflammation in the brain [45]. Finally, we used molecular docking to verify the association between active ingredients and key targets, and the results of molecular docking were consistent with the predicted interactions between targets and ingredients.

Systems pharmacology provided a robust approach to exploring the complex interactions between the ingredients and targets of KXD. In KXD, Acorus tatarinowii combined with Pinellia ternata dissolves turbidity and dispels phlegm, while Gastrodia elata and Uncaria pacify the liver and calm the wind, and Panax notoginseng combined with Pheretima has a meridian-clearing effect. This study revealed that different herbs have the same composition and that different components can act on the same target, allowing the individual herbs in KXD to better exert synergistic or antagonistic effects.

In this study, epilepsy-associated genes with differential expression were identified using microarray datasets, and the screened hub genes were validated with both ROC curves and box plots to enhance the accuracy of the prediction results. However, our study has several limitations. First, we need a more comprehensive database of TCM target genes to make the results of the network pharmacological analysis more reliable. Second, experimental validation and multidisciplinary collaboration are still needed to understand the exact mechanism of KXD in epilepsy.

## 5. Conclusion

In this study, we explored the multicomponent, multitarget, and multichannel characteristics of KXD-mediated epilepsy treatment through network pharmacology and molecular docking. The main components of KXD for epilepsy may be quercetin, baicalin, kaempferol, yohimbine, geissoschizine methyl ether, baicalein, etc. In conclusion, KXD may exert its therapeutic effect on epilepsy through the following targets: PTGS2, MMP9, CXCL8, ERBB2, and ARG1, acting on the following pathways: neuroactive ligand-receptor interactions, arachidonic acid metabolism, IL-17, TNF, NF-kappaB, and MAPK signaling pathways. This study preliminarily illustrated the mechanism of KXD in the treatment of epilepsy, providing a theoretical basis for further experimental validation and contributing to the development of effective drugs to treat epilepsy.

## Figures and Tables

**Table 1:** Active ingredients of KXD.

Herb	ID	Molecule name	Molecular formula	CAS no.
BX: Pinellia ternata	BX1	Baicalin	C_21_H_18_O_11_	21967-41-9
	BX2	Baicalein	C_22_H_20_O_11_	491-67-8
	BX3	24-Ethylcholest-4-en-3-one	C_29_H_48_O	N/A
	BX4	Cavidine	C_21_H_23_NO_4_	32728-75-9
	BX5	Coniferin	C_16_H_22_O_8_	531-29-3
	BX6	Ephedrine	C_10_H_15_NO	299-42-3
	BX7	Coniine	C_8_H_17_N	458-88-8

DL: Pheretima	DL1	Succinic acid	C_4_H_6_O_4_	110-15-6
	DL2	Xanthine	C_5_H_4_N_4_O_2_	69-89-6
	DL3	Adenine	C_5_H_5_N_5_	73-24-5
	DL4	Valine	C_5_H_11_NO_2_	72-18-4
	DL5	Leucine	C_6_H_13_NO_2_	61-90-5

GT: Uncaria	GT1	Akuammicine	C_20_H_22_N_2_O_2_	639-43-0
	GT2	Angustidine	C_19_H_15_N_3_O	40217-50-3
	GT3	Corynantheine	C_22_H_26_N_2_O_3_	18904-54-6
	GT4	Corynoxeine	C_22_H_26_N_2_O_4_	630-94-4
	GT5	Dihydrocorynantheine	C_22_H_28_N_2_O_3_	50439-68-4
	GT6	Geissoschizine methyl ether	C_22_H_26_N_2_O_3_	60314-89-8
	GT7	Hirsuteine	C_22_H_26_N_2_O_3_	35467-43-7
	GT8	Isocorynoxeine	C_22_H_26_N_2_O_4_	51014-29-0
	GT9	Isopteropodine	C_21_H_24_N_2_O_4_	5171-37-9
	GT10	Isorhynchophylline	C_21_H_26_N_2_O_4_	144525-05-3
	GT11	Rhynchophylline	C_22_H_28_N_2_O_4_	76-66-4
	GT12	Vallesiachotamine	C_21_H_22_N_2_O_3_	5523-37-5
	GT13	Corynoxine	C_22_H_28_N_2_O_4_	6877-32-3
	GT14	Yohimbine	C_21_H_26_N_2_O_3_	146-48-5

SQ: Panax notoginseng	SQ1	Liquiritigenin	C_15_H_12_O_4_	578-86-9
	SQ2	Panaxynol	C_17_H_24_O	81203-57-8
	SQ3	Ginsenoside rh2	C_36_H_62_O_8_	78214-33-2
	SQ4	Dencichine	C_5_H_8_N_2_O_5_	5302-45-4
	SQ5	Methyl palmitate	C_17_H_34_O_2_	112-39-0
	SQ6	Panaxytriol	C_17_H_26_O_3_	N/A

SCP: Acorus tatarinowii	SCP1	8-Isopentenyl-kaempferol	C_20_H_18_O_6_	28610-31-3
	SCP2	Beta-asarone	C_12_H_16_O_3_	5273-86-9
	SCP3	Alpha-asarone	C_12_H_16_O_3_	2883-98-9
	SCP4	Methyl isoeugenol	C_11_H_14_O_2_	93-16-3

TM: Gastrodia elata	TM1	Vanillin	C_8_H_8_O_3_	121-33-5
	TM2	4-Ethoxymethylphenyl-4′-hydroxybenzylether	C_16_H_18_O_3_	77160-41-9
	TM3	Gastrodin	C_13_H_18_O_7_	62499-27-8
	TM4	4-(4′-hydroxybenzyloxy)benzyl methyl ether	C_15_H_16_O_3_	N/A
	TM5	Gastrodioside	C_20_H_24_O_8_	77162-64-2
	TM6	N6-(4-hydroxybenzyl)-adenosine	C_17_H_19_N_5_O_5_	110505-75-4
BX、GT、SQ、TM	D	Daucosterol	C_35_H_60_O_6_	474-58-8
BX、GT、SQ	B	Beta-sitosterol	C_29_H_50_O	83-46-5
BX、SQ	S	Stigmasterol	C_29_H_48_O	83-48-7
BX、SCP	C	Cycloartenol	C_30_H_50_O	469-38-5
DL、TM	P	Palmitic acid	C_16_H_32_O_2_	57-10-3
GT、SQ	Q	Quercetin	C_15_H_10_O_7_	117-39-5
GT、SCP	K	Kaempferol	C_15_H_10_O_6_	520-18-3

**Table 2:** Details of KEGG enrichment analysis.

ID	Description	*p* value	Gene ID
hsa05219	Bladder cancer	9.18E-05	ERBB2 MMP9 CXCL8
hsa04657	IL-17 signaling pathway	0.001072	PTGS2 MMP9 CXCL8
hsa04071	Sphingolipid signaling pathway	0.002116	ADORA3 ASAH1 S1PR5
hsa05206	MicroRNAs in cancer	0.00413	CDC25B ERBB2 PTGS2 MMP9
hsa04913	Ovarian steroidogenesis	0.00552	PTGS2 AKR1C3
hsa04080	Neuroactive ligand-receptor interaction	0.007162	PTGER2 PTGDR ADORA3 S1PR5
hsa00590	Arachidonic acid metabolism	0.007821	PTGS2 AKR1C3
hsa05163	Human cytomegalovirus infection	0.012503	PTGER2 PTGS2 CXCL8
hsa05215	Prostate cancer	0.018985	ERBB2 MMP9
hsa01522	Endocrine resistance	0.019356	ERBB2 MMP9
hsa05146	Amoebiasis	0.020867	ARG1 CXCL8
hsa04064	NF-kappa B signaling pathway	0.021642	PTGS2 CXCL8
hsa04668	TNF signaling pathway	0.024856	PTGS2 MMP9
hsa04010	MAPK signaling pathway	0.025435	CDC25B ERBB2 CACNA2D2
hsa04670	Leukocyte transendothelial migration	0.025689	ITK MMP9
hsa04936	Alcoholic liver disease	0.038502	CPT1B CXCL8
hsa04921	Oxytocin signaling pathway	0.044611	CACNA2D2 PTGS2
hsa00220	Arginine biosynthesis	0.047584	ARG1
hsa05161	Hepatitis B	0.048873	MMP9 CXCL8

**Table 3:** Molecular docking score (kcal/mol).

Target name	PBD ID	Compounds					
		Quercetin	Baicalin	Kaempferol	Yohimbine	Baicalein	Geissoschizine methyl ether
ARG1	3THJ	−7.9	−8.3	−7.7	−6.9	−7.9	−6.5
PTGS2	5IKT	−9.6	−10.1	−9.4	−9.2	−9.2	−7.3
MMP9	5TH6	−7.4	−8.1	−7.3	−8.1	−8.7	−7.4
CXCL8	1IKL	−5.3	−6.9	−5.6	−6.4	−5.8	−6.5
ERBB2	1MFL	−5.8	−6.6	−5.6	−5.7	−5.8	−5.8

## Data Availability

All data obtained or analyzed in this study are included in the article.
